# Improvement of native structure-based peptides as efficient inhibitors of protein-protein interactions of SARS-CoV-2 spike protein and human ACE2

**DOI:** 10.3389/fmolb.2022.983014

**Published:** 2022-09-28

**Authors:** Norbert Odolczyk, Joanna Klim, Małgorzata Podsiadła-Białoskórska, Maria Winiewska-Szajewska, Ewa Szolajska, Urszula Zielenkiewicz, Jarosław Poznański, Piotr Zielenkiewicz

**Affiliations:** ^1^ Institute of Biochemistry and Biophysics, Polish Academy of Sciences, Warszawa, Poland; ^2^ Laboratory of Systems Biology, Institute of Experimental Plant Biology and Biotechnology, Faculty of Biology, University of Warsaw, Warsaw, Poland

**Keywords:** SARS-CoV-2, COVID-19, inhibitors of protein-protein interactions, peptides, drug design, angiotensin-converting enzyme-2, ACE2, coronavirus

## Abstract

New pathogens responsible for novel human disease outbreaks in the last two decades are mainly the respiratory system viruses. Not different was the last pandemic episode, caused by infection of a severe acute respiratory syndrome coronavirus 2 (SARS-CoV-2). One of the extensively explored targets, in the recent scientific literature, as a possible way for rapid development of COVID-19 specific drug(s) is the interaction between the receptor-binding domain of the virus’ spike (S) glycoprotein and human receptor angiotensin-converting enzyme 2 (*h*ACE2). This protein-protein recognition process is involved in the early stages of the SARS-CoV-2 life cycle leading to the host cell membrane penetration. Thus, disrupting this interaction may block or significantly reduce the infection caused by the novel pathogen. Previously we have designed (by *in silico* structure-based analysis) three very short peptides having sequences inspirited by *h*ACE2 native fragments, which effectively bind to the SARS-CoV-2 S protein and block its interaction with the human receptor. In continuation of the above mentioned studies, here we presented an application of molecular modeling approach resulting in improved binding affinity of the previously proposed ligand and its enhanced ability to inhibit meaningful host-virus protein-protein interaction. The new optimized hexapeptide binds to the virus protein with affinity one magnitude higher than the initial ligand and, as a very short peptide, has also great potential for further drug development. The peptide-based strategy is rapid and cost-effective for developing and optimizing efficient protein-protein interactions disruptors and may be successfully applied to discover antiviral candidates against other future emerging human viral infections.

## 1 Introduction

Recent decades have shown that viruses of wild animal origin, which break a cross-species transmission barrier, severely threaten human populations, leading to global economic, social, and healthcare problems (Baker et al., 2022). Pathogens responsible for novel human disease outbreaks in the last two decades are mainly the respiratory system viruses like severe acute respiratory syndrome coronavirus 1 (SARS-CoV-1), influenza virus A H1N1, Middle East respiratory syndrome coronavirus (MERS-CoV), and recently responsible for still ongoing pandemic a severe acute respiratory syndrome coronavirus 2 (SARS- CoV-2) (Piret and Boivin, 2021). The last pandemic episode caused by SARS-CoV-2 started in December 2019 in China (Zhu et al., 2020), has shown that new viruses are a serious challenge for the scientific community, public healthcare systems, and pharmacological companies, creating the need to rapidly react to overcome problems with lack of well-established treatments, and effective therapeutics. Despite the fact of considerable acceleration in the development and production of SARS-CoV-2 vaccines (Corbett et al., 2020), COVID-19 (coronavirus disease-2019) caused almost 6.5 million deaths in the worldwide human population until today (World Health Organization, 2020).

Thus, new technologies/strategies for the rapid development of therapeutics of novel protein pathogens targets are needed for an effective response to future pandemics (Pardi and Weissman, 2020). Among the two emerging promiscuous approaches, which may significantly reduce the time to develop new treatments in case of novel pathogens, are the drug repurposing approach (Pushpakom et al., 2019) and peptide-based therapeutics mainly targeting protein-protein interactions (Shah et al., 2022; Sharma et al., 2022).

One of the extensively explored targets to develop COVID-19 specific drug(s) (Shah et al., 2022) is an interaction between the receptor-binding domain (RBD) of the virus’ spike (S) glycoprotein (Wrapp et al., 2020) and human receptor angiotensin-converting enzyme 2 (ACE2) (Walls et al., 2020). This protein-protein recognition process is involved in the early stages of the SARS-CoV-2 life cycle leading to the host cell membrane penetration and finally the virus replication (Yan et al., 2022). Thus, disrupting the *h*ACE2 and S proteins interaction may lead to blocking or significantly reducing the infection caused by the novel pathogen, and it is worth considering as a new strategy to develop potential therapeutics against SARS-CoV-2.

The growing interest in searching/designing peptides of therapeutic value results from the increasing number of peptides already registered by FDA as drugs (currently over 60) which, in turn, results from improvements of methodologies in identifying peptidic drug candidates including phage display, high throughput screening, structure-based design and *in silico* approaches (M.C. Meireles and Mustata, 2011; Nevola and Giralt, 2015; Apostolopoulos et al., 2021). Peptides derived from native protein–protein interaction (PPI) sites of interacting partners are valuable starting points in developing effective inhibitors of protein–protein interactions, and have already been proven to be useful for the design of efficient PPI modulators (Wójcik and Berlicki, 2016; Roy et al., 2018). This, structure-based approach, where one starts with identification of continuous structure segments on experimentally known structure of the protein-protein interface is the fastest starting point to select substances for further optimisation. This is why the first, and until now the majority, of inhibitors designed against SARS-CoV-2 infection are peptides derived from the known ACE2-S complex structure [e.g. (Baig et al., 2020; Barh et al., 2020; Huang et al., 2020; Jaiswal and Kumar, 2020; Sitthiyotha and Chunsrivirot, 2020)]. The comprehensive recent review (Dahal et al., 2022) describes both peptides and peptidomimetics as therapeutic agents for COVID-19. The *in-silico* perspective on peptide-based strategies has been recently updated by Moroy and Tuffery (Moroy and Tuffery, 2022).

The protein-protein recognition process between the receptor-binding domain (RBD) of the virus’ spike (S) glycoprotein and human receptor angiotensin-converting enzyme 2 (ACE2) is involved in the early stages of the SARS-CoV-2 life cycle leading to the host cell membrane fusion and finally the virus replication (Walls et al., 2020). Immediately after the outbreak of SARS-CoV-2, the endeavor to develop a new targeted therapy against novel coronavirus was started, and one of the extensively explored drug targets in the scientific literature is the mentioned virus-host protein-protein interaction (Ghosh and Weinberg, 2021; Shah et al., 2022; Sharma et al., 2022). Many peptides with similar properties were proposed by other groups using different strategies based on natural or synthetic peptide libraries or even similar to our approach based on different, native parts of *h*ACE2 (Baig et al., 2020; Barh et al., 2020; Chatterjee et al., 2020; Chaturvedi et al., 2020; Han and Král, 2020; Huang et al., 2020; Jaiswal and Kumar, 2020; Sitthiyotha and Chunsrivirot, 2020; Zhao et al., 2022).

Recently, by *in silico* structure-based analysis, we have designed three very short peptides having sequences inspirited by *h*ACE2 native fragments, which may effectively bind to the SARS-CoV-2 S protein. The most promising peptide named **pep1d**, containing only six amino acid residues (30-DKGNHE-35), interacts with the virus protein with K_D_ ∼210 nM and can inhibit the virus-host protein-protein interaction with IC_50_ at 3.3 mM (Odolczyk et al., 2021).

In the present study, we applied an *in silico* peptide optimization approach followed by microscale thermophoresis and ELISA evaluation assays leading to the discovery of a new peptide with significantly increased binding affinity to its molecular target. Moreover, the newly identified molecule seems to be a good candidate for further drug development as it consists only of six amino acid residues, is able to effectively inhibit interaction between SARS-CoV-2 S protein and *h*ACE2 receptor, as also seems to be not toxic for human bronchial epithelial cells (BEAS 2B).

We also hope that presented a novel, rapid, and cost-effective peptide-based strategy for the development of efficient protein-protein interaction disruptors may be successfully applied to discover antiviral candidates against SARS-CoV-2 or other future emerging human viral infections.

## 2 Materials and methods

### 2.1 Molecular modeling optimization

The initial structure of the complex of SARS-CoV-2 S protein (residues 329–529) with the six-residue fragment derived from *h*ACE2 (**pep1d**: 30-DKFNHE-35) was adopted from the Protein Data Bank (www.rcsb.org) record (PDB ID: 6M0J) (Lan et al., 2020), accordingly to the previously reported analysis (Odolczyk et al., 2021). Then the optimization procedure was divided into three steps repeated in cycles. Step 1: the side-chain packing of protein and peptide structure was tuned using ten rounds of “repairPDB” procedure implemented in the FoldX package (Delgado et al., 2019) while backbone coordinates were constrained. Step 2: *in silico* mutations were introduced to peptide structure, replacing each of six positions with any of 20 natural protein amino acid residues. Step 3: The “AnalyseComplex” module (FoldX) assessed each peptide with a new mutation to estimate the changes in the binding affinity after residue replacement. The above procedure led to the ensemble of a few peptides that displayed significantly better estimated binding affinities. Each of these new peptides was then subjected to (a) the next optimization cycle (Steps 1–3), and (b) experimental binding verification. The structural visual assessment as protein structure figures were done by PyMOL software (Version 2.3.0, Schrödinger, LLC, New York, NY, United States, https://pymol.org).

### 2.2 RBD of SARS-CoV-2 protein, *h*ACE2 protein and peptides

Recombinant SARS-CoV-2 protein S1 subunit RBD domain (Arg319-Phe541) expressed in HEK293 cells (Human Embryonic Kidney 293) was purchased from Genscript®/Raybiotech- Inc. cat. no. 230–30162 (purity ≥95%). The recombinant extracellular domain (NP_068576.1) of *h*ACE2 (Met1-Ser740) protein expressed in HEK293 cells with an hFc tag attached to the C-terminus was provided by SinoBiological. cat. no. SIN-10108-H02H (purity ≥95%). The peptides: DKGNHE (**pep1d**) and DYGNHE (**J3**), DYGNYE (**J3.1**), MYGNHE (**J3.2**), LYGNHE (**J3.3**), MYGNYE (**J3.4**), LYGNYE (**J3.5**) peptides were synthesized (purity ≥95%) and supplied by GenScript Biotech (Netherlands) B.V. (https://www.genscript.com/).

### 2.3 Peptide binding by microscale thermophoresis

The MST experiments were performed with a Monolith NT 115 device (NanoTemper Technologies) using Premium MO-K025 capillaries (https://nanotempertech.com/). The obtained experimental data were analyzed according to the method described before (Winiewska et al., 2017). Models implemented in the OriginLab 2020b software (https://www.originlab.com/) were modified and instead of the dissociation constant K_D_, ΔG_D_ was fitted parameter according to formula: K_D_ = e^−ΔG_D_/RT^. The so-called global fitting was used based on at least three independent MST pseudo-titration experiments using the temperature gradient-dependent thermal diffusion effect as an indicator of the interaction. The Monolith His-Tag Labeling Kit RED-tris-NTA second generation label was attached to the C-terminal histidine tag of RBD domain according to the standard protocol provided by the manufacturer. All samples were prepared in 1×PBST (0.05%) and measurements were carried out at 22°C or/and 25°C. The concentration of the labeled protein in all experiments was kept constant at 50 nM. The peptide concentrations in two component experiments varied from 200 pM to 50 µM. In three-component experiment concentration of tested peptide was kept constant at 1 mM and the concentrations of unlabeled *h*ACE2 protein varied from 30 pM to 1 µM or 2.5 µM.

### 2.4 ELISA RBD-ACE2 interaction inhibition assay

Commercially-available “COVID-19 Spike-ACE2 binding assay kit II” (RayBiotech, United States, cat. no.: CoV-ACE2S2-2) in a 96-well plate version, the wells of which were coated with the *h*ACE2 protein, was used for the ELISA test. In this assay, the total amount of *h*ACE2-bound RBD protein (with Fc-tag) is measured in the presence of the assayed peptides. To assess the amount of the RBD protein bound to *h*ACE2, a color reaction was performed using horseradish peroxidase enzyme conjugated with anti-Fc antibodies in the presence of 3,3′,5,5′-tetramethylbenzidine (TMB) substrate. The intensity of the color reaction obtained was inversely proportional to the RBD protein concentration in the wells. The experiments were carried out according to the manufacturer’s instructions. All incubations were performed at room temperature with shaking. Peptides were dissolved in reagent provided with the kit, namely 1x concentrated Assay Diluent. 100 µl of test compound (at various concentrations) mixed with the RBD protein was added to each well. The plate was then incubated for 2.5 h. After a series of washes, 100 µl of horseradish peroxidase-conjugated anti-Fc antibody solution was added and incubated again. After another series of washes, 100 µl of TMB One-Step Substrate Reagent was added and incubated (protected from light) for another 30 min. The reaction was stopped by adding 50 µl of Stop Solution. Immediately after stopping the reaction, the absorbance was measured (at 450 nm) using a VarioskanLUX plate reader (ThermoFisher Scientific, United States).

### 2.5 Cells

BEAS 2B (cat. no.: ATCC® CRL-9609™) and Calu-3 (cat. no.: ATCC HTB-55™) cell lines were purchased from the American Type Culture Collection (ATCC, United States). BEAS 2B cells were maintained in Bronchial Epithelial Cell Growth Medium (BEGM) containing Bronchial Epithelial Basal Medium (BEBM cat. no.: CC-3171) supplemented with growth factors (SingleQuotsTM Supplement Pack cat. no.: CC-4175), both from Lonza (Switzerland). Culture flasks and multi-well plates were pre-coated with a mixture of 0.01 mg/ml fibronectin (Gibco, Thermo Fisher Scientific, United States, cat. no.: PHE0023), 0.03 mg/ml bovine collagen type I (Gibco, Thermo Fisher Scientific, United States, cat. no.: A1064401) and 0.01 mg/ml bovine serum albumin dissolved in BEBM medium. Whereas Calu-3 cells were cultured in ATCC-formulated Eagle’s Minimum Essential Medium (EMEM cat. no.: 30–2003 ATTC) supplemented with 10% Fetal Bovine Serum (FBS cat. no.: 30–2020). The culture media for both cell lines were supplemented with 100 U penicillin, and 100 μg/ml streptomycin (HyClone United States cat. no.: SV30010) upon incubation of cells in the presence of the peptide. The passage number was kept below 6 for all experiments, and the cells were grown at 37°C in a humidified, 5% CO_2_ atmosphere.

### 2.6 MTS cell viability assay

The effect of **J3** peptide on the cell viability was tested *in vitro* by MTS assay, performed according to the manufacturer’s recommendations (Promega, 2012). Briefly, BEAS 2B and Calu3 cells grown in pre-coated 96-well plates (CELLSTAR®, Greiner Bio-One, Austria, cat. no.: 655180) were exposed to **J3** peptide applied at various concentrations ranging from 1 nM to 1 mM. After 72 h of incubation at 37°C, the combined solution of MTS (Promega, United States, cat. no.: G1112) and an electron coupling reagent, phenazine methosulfate (PMS, Sigma-Aldrich, Merck, Germany, cat. no.: P9625) was added to the cell culture media, to a final concentration of 333 μg/ml MTS and 25 μM PMS. Cells were then incubated for 3 h at 37°C in a humidified, 5% CO_2_ atmosphere, and the absorbance at 490 nm wavelength was measured using a spectrophotometer (Synergy HT Microplate Reader, BioTek, United States). The absorbance values recorded for cells exposed to peptide were compared to values measured for untreated control cells, regarded as 100% cell viability.

## 3 Results

### 3.1 Optimization of native structure-based *h*ACE2 peptides

**FIGURE 1:**
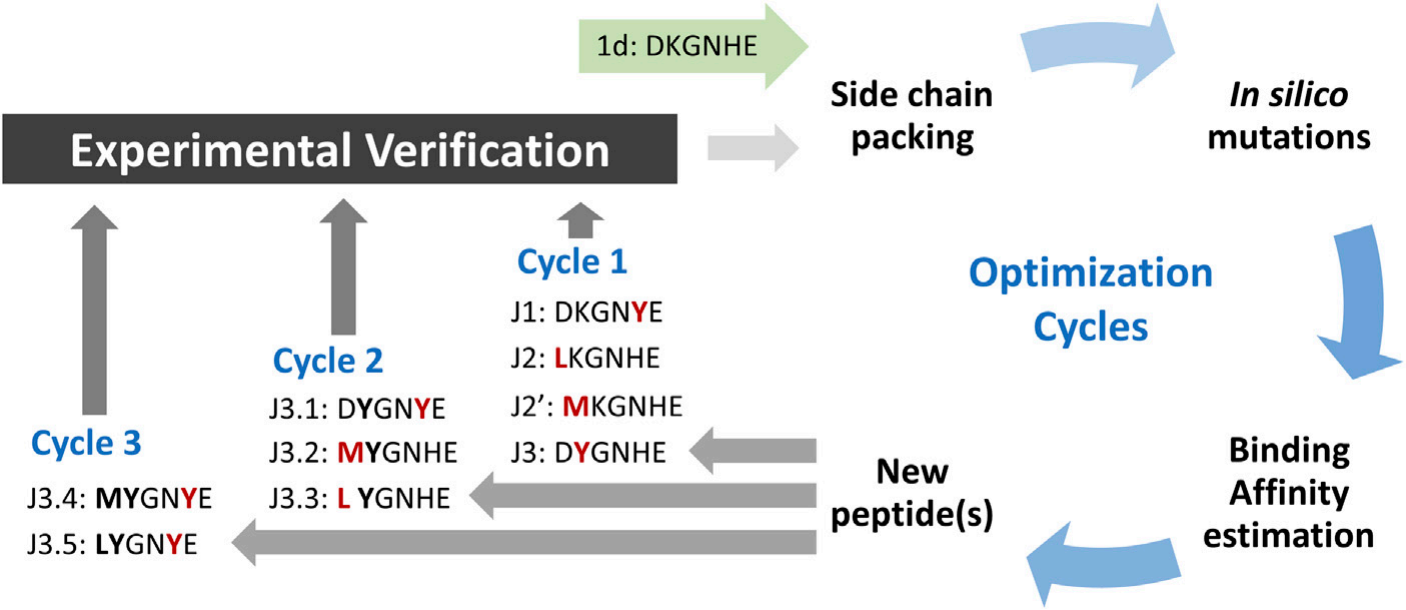
Scheme of peptide ligands optimization procedure. The amino acid residues with most favored changes in ΔG_bind_ for each run are shown in red, and bold fonts represent the advantageous mutation(s) adopted from the previous run.

As has been shown previously, the native fragment of *h*ACE peptide (with only one mutation introduced to non-interacting residue) may bind effectively to the surface of SARS-CoV-2 S protein with K_D_ ∼210 nM (Odolczyk et al., 2021). Despite a strong binding to the molecular target, its ability to inhibit host-pathogen protein-protein interaction is rather poor, as it achieved IC_50_ at about 3.3 mM. Such value leaves space for further optimization. Thus, we applied an *in silico* modeling procedure presented in Figure 1 to increase the binding affinity by modifying a particular position in the peptide sequence.

The initial complex structure with RBD domain of S protein and starting peptide **pep1d** was first optimized by side-chain packing (Step 1). Ten rounds of the FoldX module optimization approach were followed by *in silico* mutations of the peptide (Step 2). In every six positions, the starting peptide was substituted by each of the natural 20 protein amino acid residues. After each modification, the complex structure was evaluated to estimate the changes in binding affinity after residue replacement (Step 3). Further, the peptides with a significantly greater binding affinity score were selected for (a) further optimization procedure and (b) experimental verification (Tables 1 and 2). In the second round we started the optimization procedure from the peptide **J3**, which was subjected to the cycle described above (Step 1–3), which found three new sequences **J3.1**, **J3.2** and **J3.3** with better binding score according to the FoldX scoring function. The three steps of optimization in the third round was started from peptide **J3.1** resulting in two new peptide sequences **J3.4** and **J3.5**, which should bind stronger two the protein target. After three rounds of the optimization procedure, no further advantageous changes in ΔG_bind_ were observed for the subsequent mutations, thus the procedure was aborted.

**TABLE 1:** Selected new peptides from each round of optimization to experimental verification. The most favored changes in ΔG_bind_ were shown for residue replacement indicated by square brackets. The residues in round brackets indicated the advantageous mutation(s) adopted from the previous run.

Round	Peptide ID	Sequences						ΔΔG_bind_ (kcal/mol)
		1	2	3	4	5	6	
0	pep1d	D	K	G	N	H	E	-
1	J1	D	K	G	N	**[Y]**	E	2.1
	J2	**[L]**	K	G	N	H	E	1.5
	J2′	**[M]**	K	G	N	H	E	2.1
	J3	D	**[Y]**	G	N	H	E	1.5
2	J3.1	D	**(Y)**	G	N	**[Y]**	E	2.2
	J3.2	**[M]**	**(Y)**	G	N	H	E	0.9
	J3.3	**[L]**	**(Y)**	G	N	H	E	0.9
3	J3.4	**(M)**	**(Y)**	G	N	**[Y]**	E	0.4
	J3.5	**(L)**	**(Y)**	G	N	**[Y]**	E	0.5

**TABLE 2:** Binding affinity of the tested peptides (and the reference initial peptide **pep1d**: DKGNHE) to RBD domain of the SARS-CoV-2 S protein determined with the aid of MST. The two different temperatures were applied to be able to compare results with the previously reported values (Odolczyk et al., 2021).

Peptide ID	Sequence	K_D_ (nM) T_measur._ 22°C	K_D_ (nM) T_measur._ 25°C
pep1d	DKGNHE	210 ± 50^a^ 238 ± 34	278 ± 39
J3	DYGNHE	42 ± 8	50 ± 10
J3.1	DYGNYE	121 ± 37	142 ± 43
J3.2	MYGNHE	83 ± 20	98 ± 24
J3.3	LYGNHE	162 ± 23	190 ± 26
J3.4	MYGNYE	Nd	>10 000
J3.5	LYGNYE	Nd	>1 000

a value reported previously (Odolczyk et al., 2021)

### 3.2 Binding of the peptides to the viral S glycoprotein (microscale thermophoresis)

To confirm that the optimized peptide inhibitors can bind to the surface of viral S glycoprotein Microscale Thermophoresis (MST) method was applied (Table 2, Supplementary Figures S1–S5). The binding affinity of each peptide towards the RBD domain of the S1 subunit of viral S glycoprotein was measured. The estimated dissociation constants (K_D_) of the peptides and the RBD domain of the SARS-CoV-2 S protein complexes are presented in Table 2. Peptides with the sequence DYGNHE (**J3**) and MYGNHE (**J3.2**) from the first and second cycle respectively, exhibit significantly higher binding affinity than the native peptide **pep1d** proposed in the previous studies (Odolczyk et al., 2021).

### 3.3 *In vitro* RBD-*h*ACE2 interaction inhibition assays

In a further step, to verify whether the proposed peptides, after binding to the surface of the viral protein, can block its interaction with the *h*ACE2 receptor, three-component experiments were performed in the system of the RBD domain of the S protein, *h*ACE2 receptor, and the peptide.

#### 3.3.1 *In vitro* RBD-*h*ACE2 interaction inhibition assay (ELISA)

**FIGURE 2:**
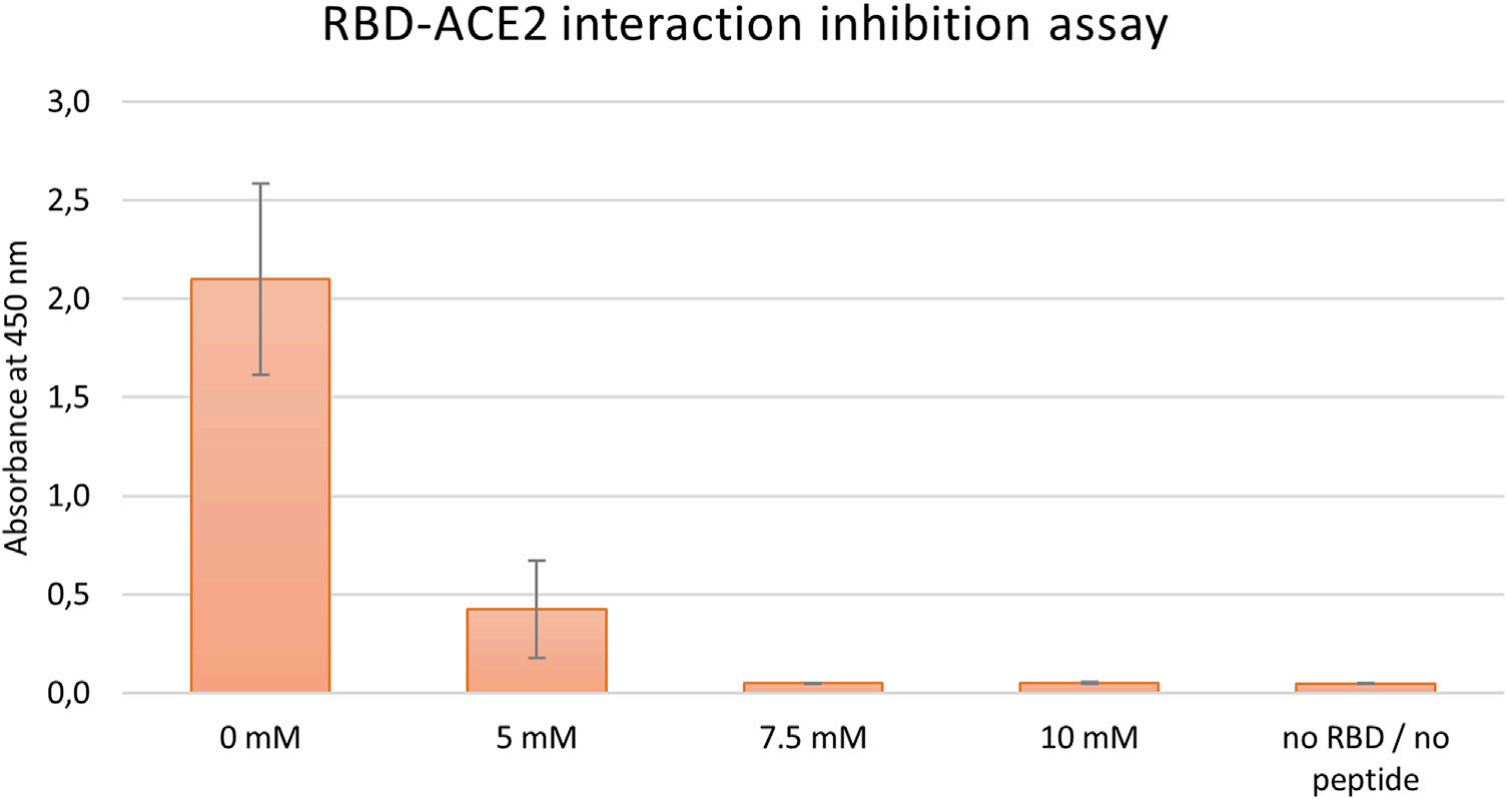
Inhibition of SARS-CoV-2-S-RBD binding to *h*ACE2 by various concentrations of the peptide J3. Graph represents data from three independent biological replicates.

Initially, the inhibitory effect of tested peptides was monitored by a commercial COVID-19 Spike-*h*ACE2 binding assay kit II (RayBiotech), as described in the Material and Methods section. First, in the preliminary run peptides **J3, J3.2**, and **J3.3** were examined. Conducted experiments showed that the most promising results were obtained for **J3** peptide only (data not shown). Thus, this peptide was subjected further to the dose-dependent inhibition run. As has been shown in Figure 2 the dose-dependent inhibition of RBD-*h*ACE2 interaction was observed for **J3** concentrations higher than 5 mM resulting in almost complete blocking of the RBD-ACE2 interaction. ELISA test has proved that the **J3** peptide has inhibitory activity and is likely to interfere with the S protein−*h*ACE2 binding needed for viral attachment.

#### 3.3.2* In vitro* RBD-*h*ACE2 interaction inhibition assay (microscale thermophoresis)

First, two-component measurements were performed to estimate the dissociation constant K_D_ of the complex of the RBD domain of the S protein (fluorescently labeled) with the *h*ACE2 protein. The experiment was then repeated in the presence of the appropriate peptide at a constant final concentration of 1 mM, to verify if the peptide blocks the interaction between the proteins.

**FIGURE 3:**
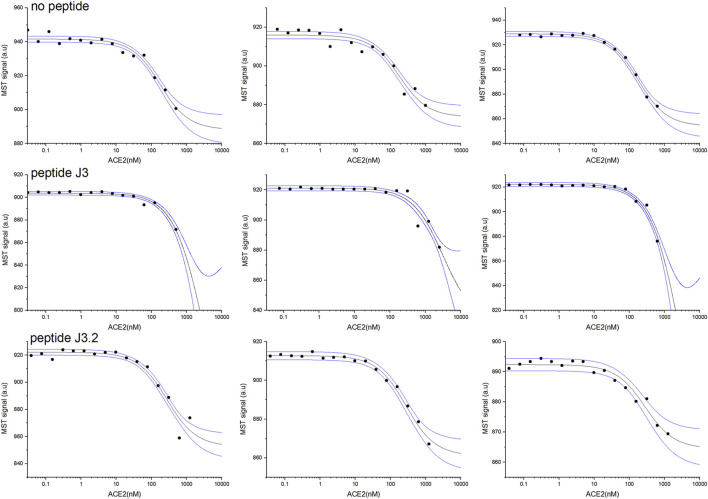
RBD and *h*ACE2 interaction inhibition assay for best peptides binders J3 and J3.2 monitored by MST pseudo-titration experiments. Two components experiment without peptides (upper panel). Three component experiment repeated in the presence of peptide J3 (middle panel) and peptide J3.2 (lower panel) at a constant concentration of 1 mM. The concentration of *h*ACE2 (unlabeled) varied from 30 pM to maximum 2.5 µM, whereas RBD (His-Tagged) was kept constant at 50 nM. Black lines represent the model fitted for each peptide globally using data from at least three independent MST pseudo-titration experiments, while blue lines denote the 95% confidence bands for the fitted line.

As shown in Table 3, the measured dissociation constant (K_D_) for the RBD-*h*ACE2 complex is 151±23 nM which is in line with previously reported values of 133.3±5.6 nM (Wang et al., 2020). The addition of DYGNHE (**J3**) peptide significantly increase the dissociation constant to ∼720 nM, while in presence of MYGNHE (**J3.2**) peptide dissociation constant increased only to ∼253 nM (Figure 3/Table 3). Other peptides bind to the RBD domain with the affinity comparable to *h*ACE2 so one may assume much worse inhibitory potency.

**TABLE 3:** Dissociation constants of the RBD-*h*ACE2 complex determined on the basis of the MST experiments with and without tested peptide.

Peptide ID	Sequence	K_D_ (nM)T_measur._ 25°C
without peptide	-	151 ± 23
J3	DYGNHE	720 ± 115
J3.2	MYGNHE	253 ± 49

### 3.4 Viability of human bronchial epithelial cells upon *in vitro* exposure to the J3 peptide

Considering the safety of future application of promising peptide candidate as therapeutic against SARS-CoV-2 infection, the potential peptide toxicity was evaluated using a human cell-based *in vitro* assay. Since the proposed peptide is intended to act in the respiratory tract, the noncancerous bronchial epithelial BEAS 2B and the lung cancer Calu-3 cell lines were chosen for cytotoxicity testing.

The effect of the **J3** peptide on the BEAS 2B and Calu-3 cells was assessed by MTS assay, which is based on the conversion of 5-(3-carboxymethoxyphenyl)-2-(4,5-dimethylthiazoly)-3-(4-sulfophenyl) tetrazolium inner salt into colored aqueous soluble formazan, carried out by dehydrogenase enzymes found in metabolically active cells. The amount of formazan product measured spectrophotometrically is directly proportional to the number of viable cells (Berridge et al., 2005).

**FIGURE 4:**
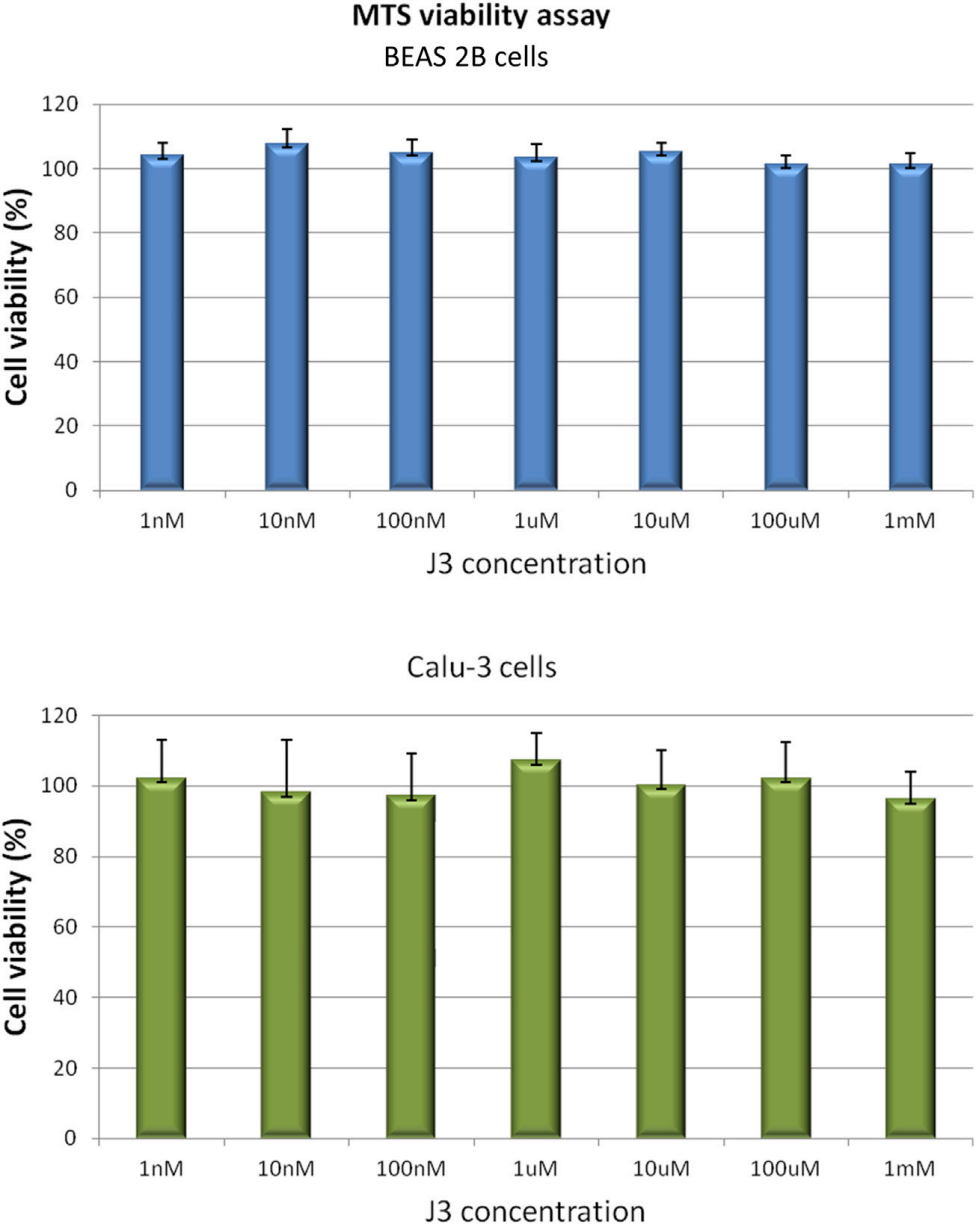
BEAS 2B and Calu-3 cells grown in a 96-multiwell plates were exposed for 72 h to **J3** peptide applied at various concentrations ranging from 1 nM to 1 mM. Cell viability was determined by MTS assay. Values are represented as mean ± SD of three independent experiments.

As demonstrated in Figure 4 the viability of BEAS 2B and Calu-3 cells was not affected upon the 72 h of exposure to the peptide applied at concentrations up to 1 mM. However, it has to be noticed, that cell membrane penetration by the peptide was not tested in our assay. Thus, not observable toxicity could be also an effect of peptide retention outside the cell.

## 4 Discussion

Our previous *h*ACE2 native structure-based approach to develop the effective protein-protein interaction inhibitor resulted in three peptide ligands, of which the best **pep1d** interacts with the S viral protein with the affinity at K_D_ ∼210 nM. In the current contribution, we presented an approach leading to optimized sequence of the peptide proposed previously which increased its binding to molecular target significantly (K_D_ ∼50 nM).

**FIGURE 5:**
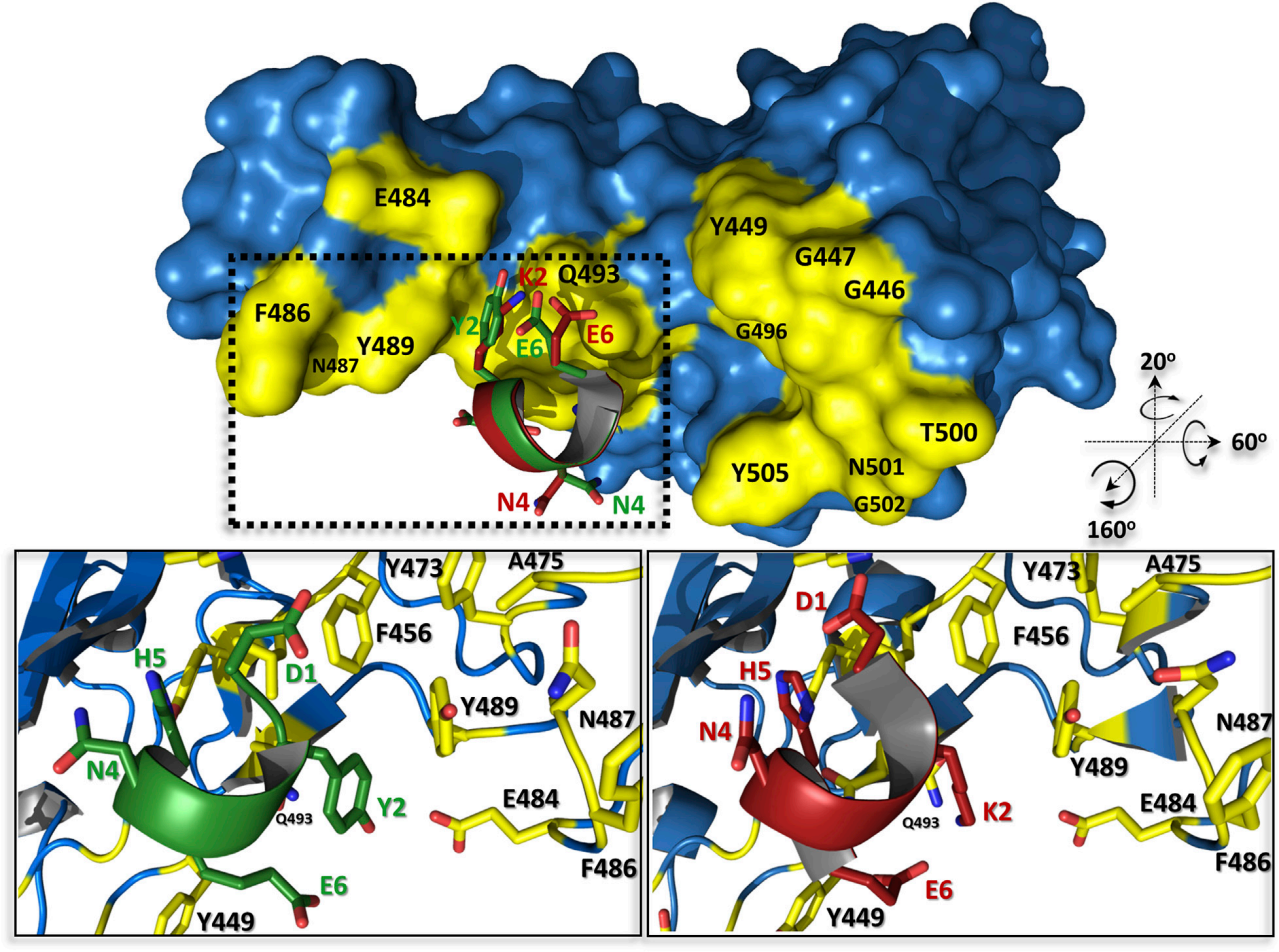
The predicted complex structures of peptides pep1d (red) and J3 (green) with SARS-CoV-2 spike receptor-binding domain (blue). Residues creating interaction interface area with *h*ACE2 are coloured yellow. Detailed view of interactions between pep1d (lower right panel) and J3 (lower left panel).

This change is caused only by one substitution at the second position of the peptide, where the positively charged lysine has been replaced by the neutral hydrophobic tyrosine (Figure 5). From a structural point of view, it could be explained by several new types of interactions created with a surrounding protein: 1) tyrosine much more tightly fits the hydrophobic groove formed by L455, F456, and Y486; 2) its hydroxyl group can be a donor of hydrogen bond with the side chain atoms of E484, as also 3) may participate in an amino-aromatic interaction with Q493 as their distance and geometry fit perfectly to such kind of contact observed in proteins (Burley and Petsko, 1986).

Interestingly among all proposed mutations of pep1d, only one had a significant positive impact on peptide binding affinity. Such a situation could be caused by several reasons like 1) imperfections of the used force field and the binding scoring function, 2) entropy penalties during binding of different sequences, as also 3) by slightly different binding modes of peptides selected for further optimization. Thus, it is worth mentioning that only the structure of **pep1d** was adopted from the crystal structure, whereas the other peptides’ binding modes were derivative of the optimization procedure used in the studies.

It is worth mentioning that our peptide **J3** is one of the shortest peptides currently known, which binds to the S protein of SARS-CoV-2 with such high affinity. Peptides as a class of molecules are characterized by low metabolic stability, unfavorable pharmaco-kinetics, poor cell membrane permeability, or even undesired immune host system response. Thus, such a short peptide may have a greater chance of overcoming all the above drawbacks and are suitable for further drug development. We consider that the **J3** peptide meets the important requirement for a drug candidate, which is the lack of cytotoxicity. However, it should be highlighted that cell membrane penetration by the peptide was not tested in our assay. Thus, not observable toxicity could be also an effect of peptide retention outside the cell, which could be also a beneficial situation from a pharmacological point of view. Therefore, the peptide could be administrated as a drug directly to the lungs and respiratory tract via inhalation, where it should act on extracellular space to effectively block the virus entry.

In conclusion, the results of this study have provided potential candidates for further development of therapeutics against SARS-CoV-2 infection. However, we also hope that the presented rapid, cost-effective peptide-based strategy for developing and optimizing efficient protein-protein interaction disruptors may be successfully applied to discover antiviral candidates against future emerging human viral infections (Buchwald, 2022).

## Data Availability

The raw data supporting the conclusion of this article will be made available by the authors, without undue reservation.
