# Supplementary material for: Improvement of native structure-based peptides as efficient inhibitors of protein-protein interactions of SARS-CoV-2 spike protein and human ACE2
Source: Front Mol Biosci. 2022 Sep 28;9:983014. doi: 10.3389/fmolb.2022.983014 (PMC9555309; doi:10.3389/fmolb.2022.983014)
Supplement: Supplementary file 1 [file DataSheet1.PDF]

# *Supplementary Material*

## 1 Supplementary Figures

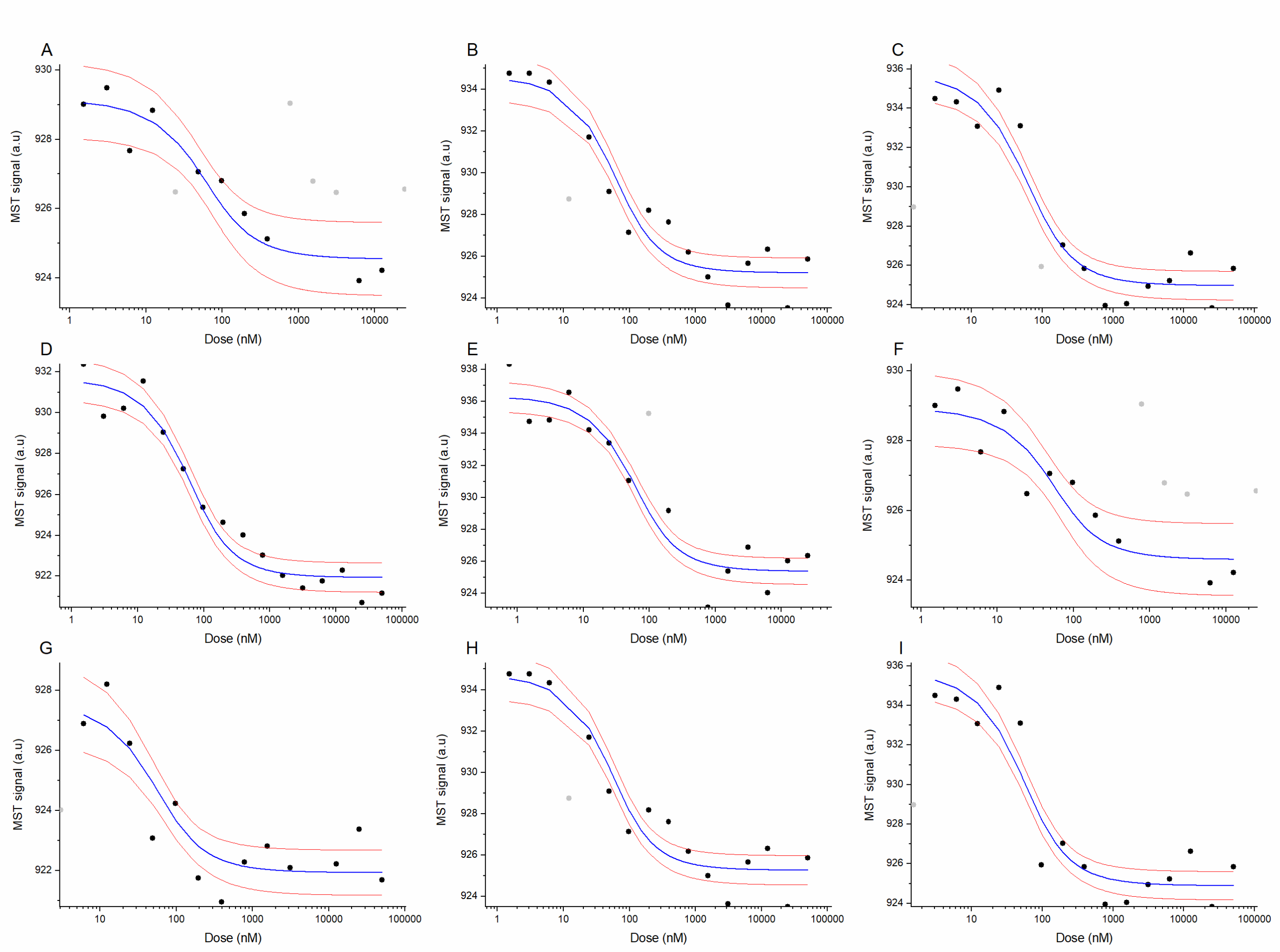


**Supplementary Figure S1.** Interaction of RBD domain of SARS-CoV-2 protein with peptides, monitored with MST pseudo-titration experiments. Blue lines represent the model fitted for peptide J3 globally, while red lines denoted the 95% confidence bands for the fitted line. Gray circles identify data excluded from the analysis.

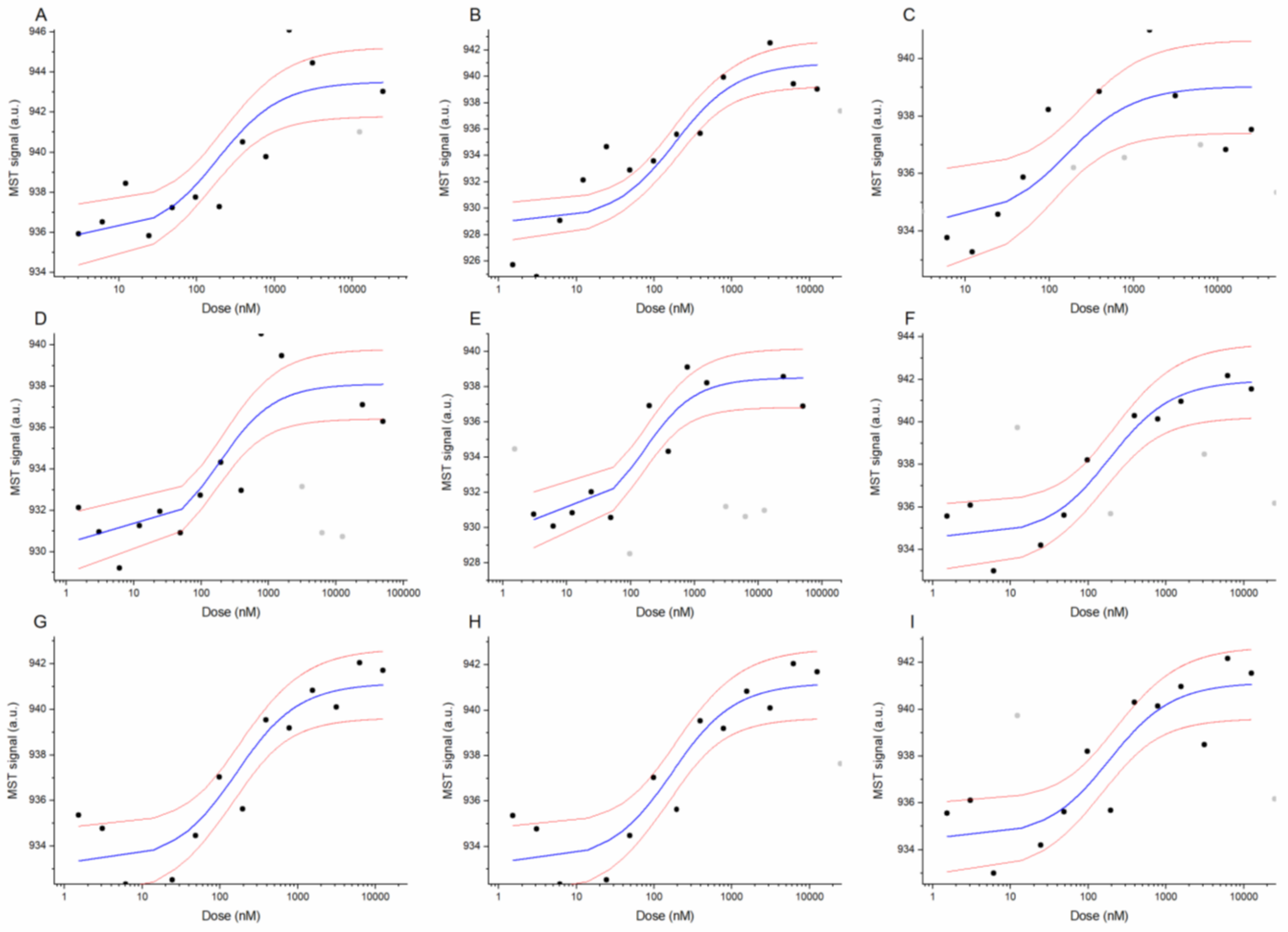


**Supplementary Figure S2.** Interaction of RBD domain of SARS-CoV-2 protein with peptides, monitored with MST pseudo-titration experiments. Blue lines represent the model fitted for peptide J3.1 globally, while red lines denoted the 95% confidence bands for the fitted line. Gray circles identify data excluded from the analysis.

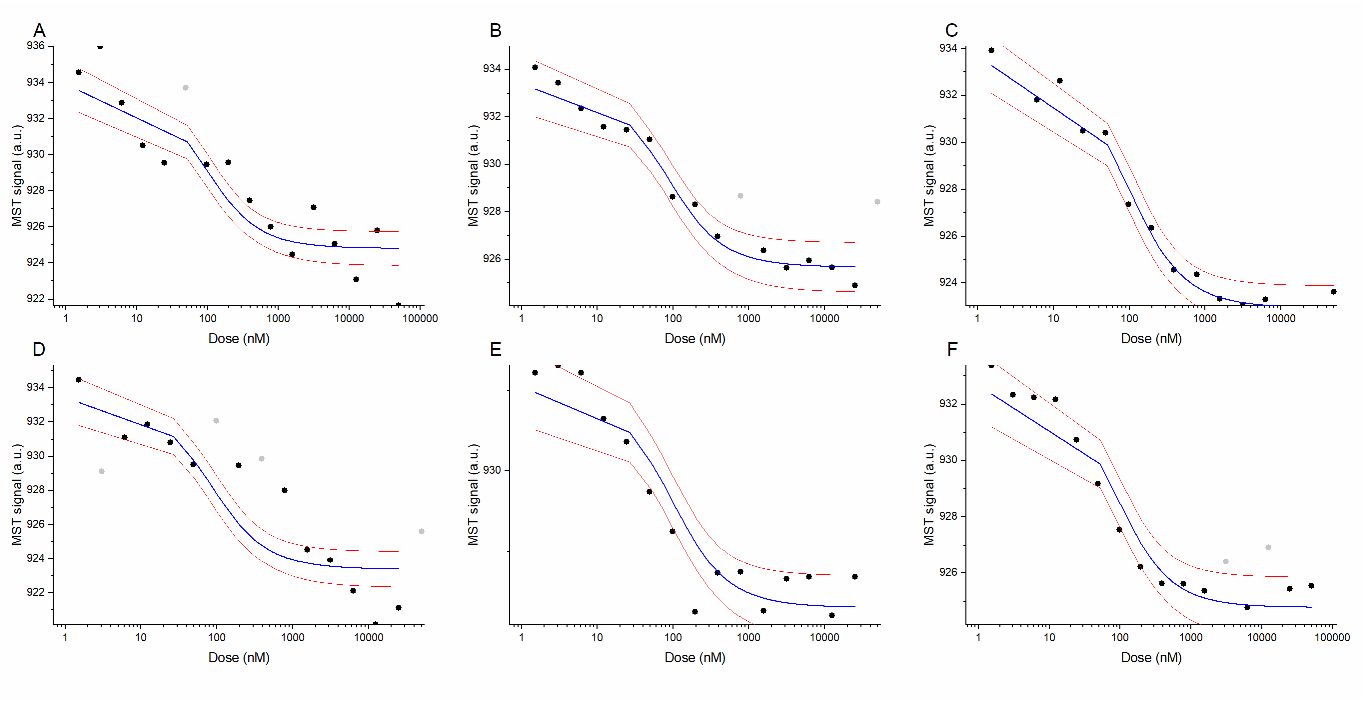


**Supplementary Figure S3.** Interaction of RBD domain of SARS-CoV-2 protein with peptides, monitored with MST pseudo-titration experiments. Blue lines represent the model fitted for peptide J3.2 globally, while red lines denoted the 95% confidence bands for the fitted line. Gray circles identify data excluded from the analysis.

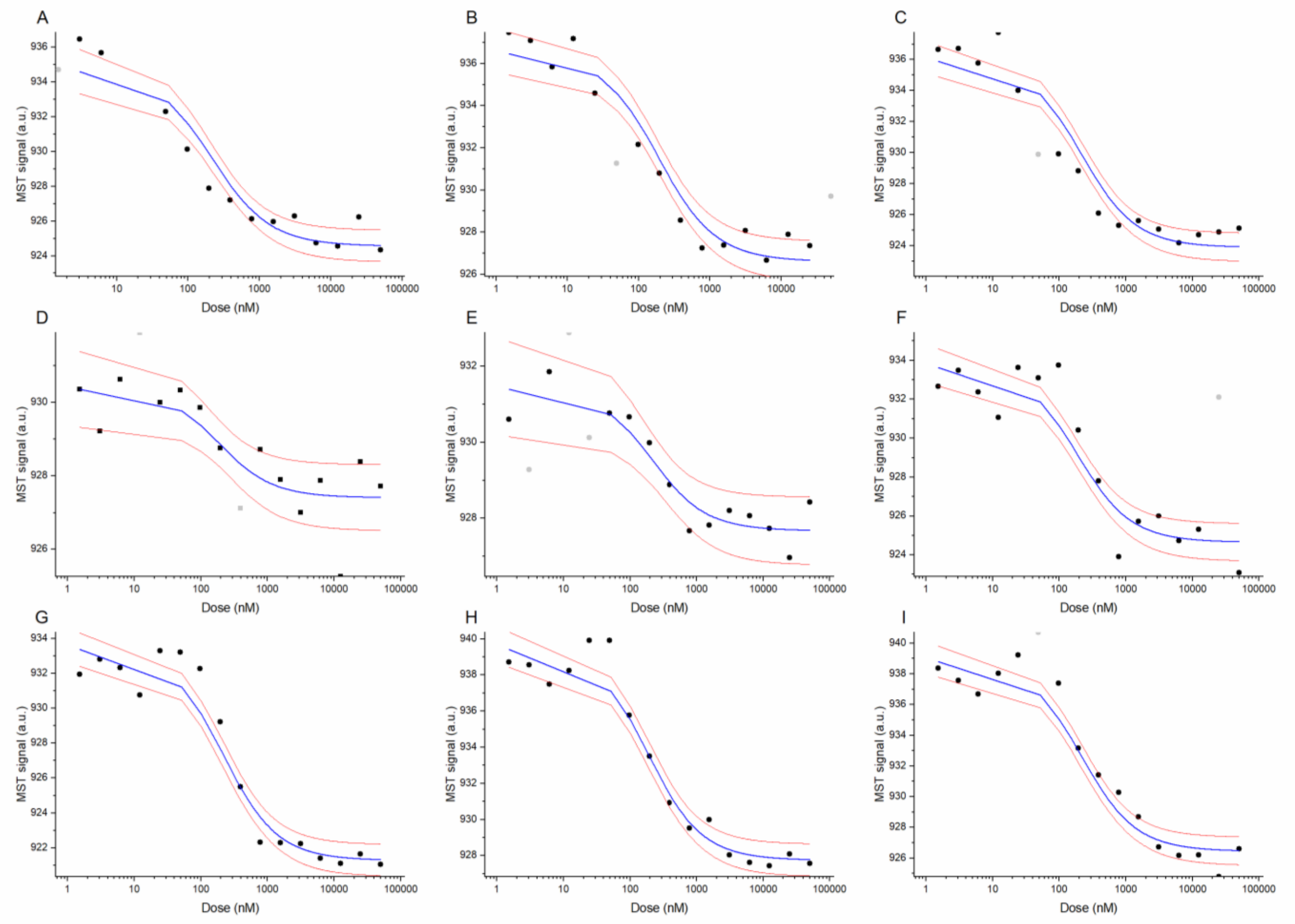


**Supplementary Figure S4.** Interaction of RBD domain of SARS-CoV-2 protein with peptides, monitored with MST pseudo-titration experiments. Blue lines represent the model fitted for peptide J3.3 globally, while red lines denoted the 95% confidence bands for the fitted line. Gray circles identify data excluded from the analysis.

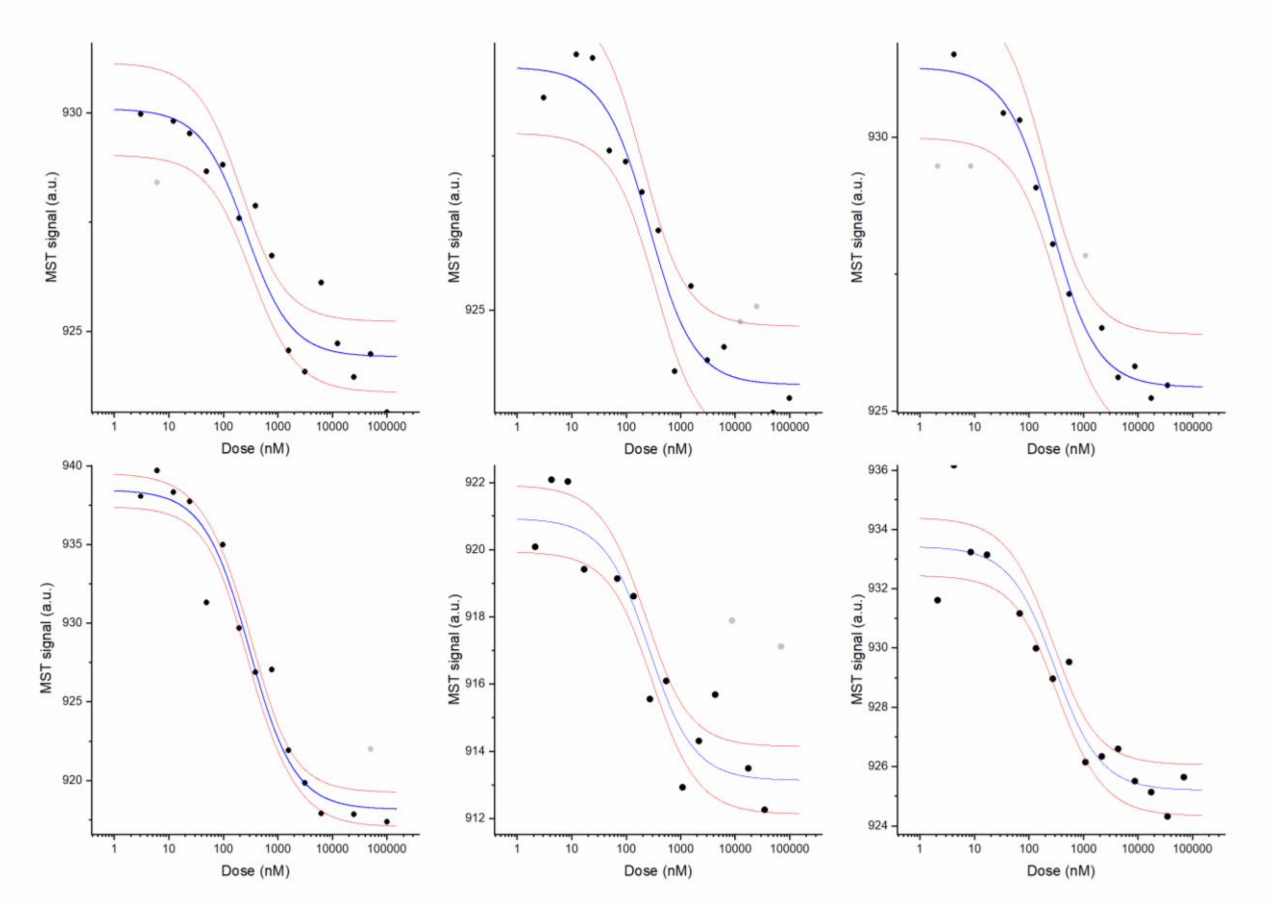


**Supplementary Figure S5.** Interaction of RBD domain of SARS-CoV-2 protein with peptides, monitored with MST pseudo-titration experiments. Blue lines represent the model fitted for peptide pep1d globally, while red lines denoted the 95% confidence bands for the fitted line. Gray circles identify data excluded from the analysis.
